# Diabetic cardiomyopathy: a comprehensive review of diagnosis, management, and future directions

**DOI:** 10.1186/s13098-025-01986-0

**Published:** 2025-11-10

**Authors:** Bhagyalakshmi Balakrishnan, Divyashree M. Somashekara, Veena Nayak, Raghu Chandrashekar Hariharapura

**Affiliations:** 1 Department of Biotechnology, Manipal Institute of Technology, Manipal Academy of Higher Education, Manipal, 576104 Karnataka India; 2 Department of Pharmacology, Kasturba Medical College, Manipal Academy of Higher Education, Manipal, 576104 Karnataka India; 3 Department of Pharmaceutical Biotechnology, Manipal College of Pharmaceutical Sciences, Manipal Academy of Higher Education, Manipal, 576104 Karnataka India

**Keywords:** Diabetic cardiomyopathy, Heart failure, Ventricular dysfunction, Hypertrophy, GLP1, SGLTi

## Abstract

Diabetic cardiomyopathy is a growing challenge to global public health. The clinical characteristics are mainly fibrosis, hypertrophy, ventricular dysfunction, cardiac remodelling, and finally, heart failure. This alarming condition is often difficult to diagnose. Routine tests such as echocardiogram, electrocardiogram, and magnetic resonance imaging, along with specialized techniques such as transmitral Doppler, tissue Doppler imaging, and positron emission tomography, help diagnose and offer clearer insight into the condition. Current treatment strategies mainly address this medical condition as a condition secondary to diabetes. The main therapeutic practices involve glucose-lowering drugs such as glucagon-like peptide-1 modulators and sodium‒glucose cotransporter-2 inhibitors, along with other conventional lines of treatment to manage complications. Promising new potential therapeutic methods include gene therapy and noncoding RNA-mediated regulation of disarrayed signalling cascades. In this review, we address the benefits and limitations of the current system in the diagnosis and treatment of diabetic cardiomyopathy as well as the possibilities and advantages of novel techniques. In addition to discussing biomarkers for screening, diagnosis, and drug response for diabetic cardiomyopathy, we also discuss management and prevention strategies, adversities, and quality of life, and provide a brief outlook for research in this area.

**Graphical abstract:**

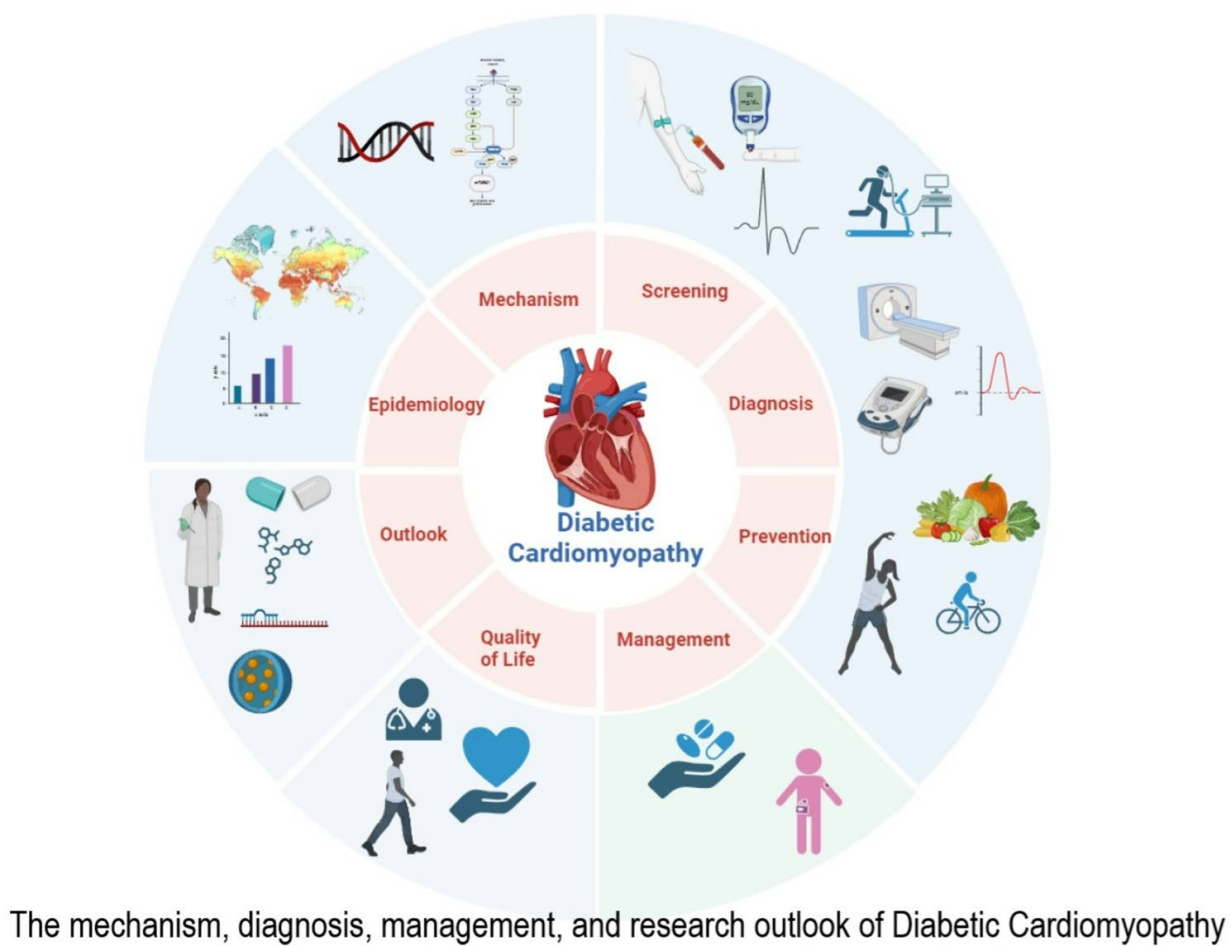

## Introduction

Diabetic cardiomyopathy (DbCM) is a condition arising from the complex interplay of metabolic disturbances. Chronic hyperglycaemia, lipotoxicity, and insulin resistance promote oxidative stress, inflammation, and advanced glycation end-products, which together impair cardiomyocyte metabolism, promote interstitial fibrosis, and alter myocardial energetics. These disturbances affect the metabolic plasticity of the heart cells. This causes less efficient energy production and oxidative stress [1–3]. To compensate for the physiological burden, the cardiac cells increase in size, which is known as hypertrophy. In addition, excessive deposition of collagen fibres, thickening of the extracellular matrix called fibrosis, and ventricular dysfunction occur in such settings [4]. The heart muscles gradually lose their contractile ability, leading to heart failure (HF) [5]. Endothelial dysfunction and coronary microvascular impairment also manifest as reduced NO bioavailability, endothelial-to-mesenchymal transition, and impaired coronary flow reserve. This also links systemic metabolic disturbance to myocardial ischemia, fibrosis, and eventual systolic/diastolic failure [6].

Jankauskas et al. (2021) reviewed the cellular mechanisms underlying DbCM, highlighting the contributions of cardiomyocytes, fibroblasts, endothelial cells, and smooth muscle cells. They emphasized that bioenergetic disturbances, oxidative stress, and myocardial fibrosis are key drivers in the progression of heart failure in patients with diabetes [7]. Xiang et al. (2024) identified the PACS2/CPT1A/DHODH signaling pathway as a key driver of DbCM. This cascade promotes cardiomyocyte ferroptosis via mitochondrial dysfunction, where PACS2 downregulation reduces CPT1A expression, elevates DHODH activity, and triggers iron accumulation with lipid peroxidation. These findings mark a paradigm shift in understanding DbCM cell death mechanisms and highlight PACS2 modulation as a promising therapeutic target and potential biomarker, since its inhibition effectively reversed cardiac dysfunction and ferroptosis in experimental models [8]. Li et al. (2023) identified ATF4 as a critical transcription factor driving diabetic cardiac fibrosis through a novel ATF4/Smurf2/HIPK2/Nrf2 signaling axis [9]. These mechanisms underlying the pathophysiology provide crucial insights for therapeutic research.

Although preclinical models have illuminated many DbCM mechanisms, direct human myocardial data provide critical translational insight. Marfella et al. (2020) performed heterotopic heart transplants from nondiabetic donors into diabetic recipients, demonstrating marked myocardial lipid accumulation and altered phospholipid composition in human hearts exposed to diabetes in vivo, establishing lipotoxicity as a fundamental driver of DbCM [10]. Marfella et al. (2022) examined ventricular biopsies from diabetic patients treated with ACE inhibitors and found that nonenzymatic glycation of ACE2 in human diabetic hearts diminished the antifibrotic and antihypertrophic benefits of RAAS blockade. Glycated ACE2 exhibited reduced enzymatic activity and impaired angiotensin (1–7) production, correlating with persistent myocardial fibrosis and dysfunction despite ACE inhibitor therapy. These findings reveal hyperglycemia-mediated ACE2 modification as a key mechanism limiting RAAS inhibitor efficacy in human DbCM [11]. These human myocardial investigations are critical for bridging the gap between preclinical discoveries and clinical application.

Diabetic individuals have a three to five-fold increased risk of developing HF compared to non-diabetic individuals, underscoring the clinical significance of DbCM [12]. HF is a common end-stage manifestation of DbCM, often resulting from progressive myocardial dysfunction. However, diabetes remains undiagnosed in nearly 50% of cases, and DbCM is typically asymptomatic in its early stages, making early detection challenging. Swiatkiewicz et al. (2023) investigated the prevalence of DbCM among patients with type 2 diabetes mellitus (T2DM) at a large academic medical center. In this cohort study, they found that approximately 2.9% of patients met the diagnostic characteristic of DbCM [13]. Therefore, a structured and integrated approach to screening, diagnosis, and timely intervention is essential to mitigate the progression of DbCM and prevent HF.

Current therapeutic interventions for DbCM primarily target individuals with underlying diabetes via the use of the antidiabetic drugs, metformin and insulin [14]. However, tailored therapies targeting the pathophysiology of DbCM are lacking [15]. Lifestyle interventions can always reduce complications. This includes healthy, balanced food rich in fibre, whole grains, and vegetables; adequate physical activity for better blood circulation; and good sleeping habits [16]. The risk factors involved in DbCM are similar to those associated with most metabolic disorders, including overnutrition, obesity, a sedentary lifestyle, a lack of sleep, smoking, and alcohol consumption [17].

Despite growing recognition of DbCM as a distinct clinical entity, several challenges remain unresolved. A major barrier is the lack of standardized diagnostic criteria, which hampers both early detection and comparability across studies. The understanding of the management of the condition, especially the role of antidiabetic medications, insulin, metformin, and other therapies for handling treatment resistance, is essential. These gaps underscore the need for a comprehensive synthesis of available evidence to clarify current knowledge and highlight future directions. This review discusses screening, diagnosis, therapeutic management, quality of life, and possible preventive measures for DbCM. We also discuss the outlook for the coming years in recognizing DbCM as a clinical entity and exploring and expanding its management strategies.

## Screening of DbCM

DbCM screening practices are poorly defined in the clinical setting. Although research has progressed in imaging technologies and biomarker discovery, no specific and efficient screening technique has been established in practice. Echocardiography is the conventional method used to detect cardiac dysfunction and can be employed for DbCM detection [18]. However, the primary concern is the asymptomatic nature of early-stage DbCM, with symptoms often emerging only in later stages, culminating in HF. Susceptibility methods such as strain, strain rate, and myocardial tissue velocity can be used to screen for DbCM in the early stages [19]. A body mass index (BMI) above 25 kg/m^2^ is a high-risk factor for cardiometabolic complications [20]. A glycosylated Hb content above 6.5% is also a red flag [21]. It is inversely proportional to the peak late filling velocity in the heart. Pro hypertrophic biomarkers such as cardiotrophin-1, proteins with a positive correlation with cardiomyocyte contraction, such as activin A, and those associated with steatosis, such as heart fatty acid-binding protein, have great potential as markers for screening DbCM [18].

Current screening methods for DbCM are limited by heterogeneous study designs, small sample sizes, and a lack of standardized protocols, which restricts the generalizability and practical utility of proposed approaches [22–24]. While advancements in imaging and biomarkers show promise, robust validation in diverse and asymptomatic populations is still needed to establish efficient screening strategies for clinical practice.

## Diagnosis of DbCM

Imaging techniques have the ability to detect structural and functional changes happening in the heart. During the initial asymptomatic stages, an electrocardiogram (ECG) efficiently analyzes subtle changes in the heart. Exercise-induced ECG alterations may indicate the preclinical phase of DbCM. Novel techniques such as positron emission tomography (PET) can analyse the metabolic perturbations, such as increased fatty acid metabolism or reduced glucose metabolism, especially for patients with advanced left ventricular dysfunction [18]. Modalities such as molecular magnetic resonance imaging, delayed gadolinium enhancement cardiac magnetic resonance imaging, and 2D speckle tracking imaging are being explored to better diagnose disease progression [25]. Recent perspectives emphasize that DbCM represents a complex and evolving phenotype, with diverse clinical indicators and emerging therapeutic targets. Choudhury et al. (2024) highlighted these challenges in their editorial, underscoring the need for integrative approaches to diagnosis and treatment that extend beyond conventional glucose control [26]. The current clinical practices for screening and diagnosing DbCM are provided below **(**Table 1**)**.

**Table 1:** Screening and diagnosis of diabetic cardiomyopathy

Technique	Clinical parameter	Characteristic	Reference
**Physical examination**			
BMI	Risk factors for cardiac dysfunction	Non-Invasive	[20]
**Blood tests**			
HbA1c	Diastolic dysfunction	Minimally invasive	[27]
BNP	Hypertrophy	Minimally invasive	[28]
CRP	inflammation	Minimally invasive	[29]
Troponins	Hypertrophy, necrosis	Minimally invasive	[18]
**Imaging techniques**			
X-ray	Fluid accumulation in lungs	Non-Invasive	[18]
Electrocardiograph	Fibrosis	Non-Invasive	[30]
Echocardiography	Tissue velocity imaging + strain and strain rate	Non-Invasive	[31]
Tissue Doppler Imaging	Left ventricular diastolic dysfunction	Non-Invasive	[32]
Cardiac MRI	Cardiac remodelling, fibrosis, fluid accumulation, TG content, and cardiac energetics	Non-Invasive	[33]
Cardiac CT	Cardiac remodelling, Dilated LA or dilated left ventricle with diastolic dysfunction	Non-invasive	[34, 35]
PET	Oxidative stress, fibrosis, metabolic perturbations	Minimally invasive	[36–38]
Cardiac catheterization	Diastolic dysfunction	Invasive	[39]
Biopsy	Hypertrophy, fibrosis, necrosis, and pathological changes	Invasive	[39]

HbA1c, glycated haemoglobin; BMI, body mass index; BNP, brain natriuretic peptide; CRP, C-reactive protein; TG, triglyceride; MRI, magnetic resonance imaging; CT, computed tomography; LA, left atrium; LV, left ventricle; PET, positron emission tomography

### Echocardiography (Echo)

Echocardiography is the current non-invasive gold standard test for studying structural and functional cardiac disorders. It uses ultrasound, frequently more than 20,000 Hz, to examine the heart condition [40]. As the primary modality for many cardiovascular diseases, it can detect early changes in the heart, such as impaired diastolic filling or left ventricular hypertrophy [18].

While echocardiography serves as the conventional method for detecting cardiac dysfunction in DbCM screening, studies supporting this approach show considerable heterogeneity in methodology and sample sizes. Sample size limitations affect many echocardiographic studies, with some including fewer than 100 patients, potentially limiting the generalizability of findings to broader diabetic populations. Study design variability is evident, with cross-sectional studies predominating over longitudinal cohorts, restricting our understanding of DbCM progression patterns [22].

### Doppler echocardiography

Doppler echocardiography is a valuable, non-invasive tool for early detection and assessment of DbCM. Doppler technology, combined with two-dimensional echocardiography, helps to study the cardiac structure and its movement in synchronization with blood flow. It helps analyse anomalous flow, such as valve regurgitation, the severity of valve stenosis, and even epicardial adipose tissue accumulation, along with diastolic or systolic function and hypertrophy [18]. Manoeuvres such as Valsalva (forced expiration against the closed glottis) are also employed with Döppler signals for even greater accuracy [41].

Doppler echocardiography is indeed a valuable, non-invasive tool for early DbCM detection, yet its diagnostic performance can be limited by operator dependency, technical constraints, and heterogeneity in study protocols, particularly for advanced maneuvers like Valsalva, where reproducibility and signal quality may vary. Most supporting studies are small-scale or single-center, which may not fully represent diverse clinical populations, and standardized guidelines for its use in diabetes-specific cardiomyopathy screening are still lacking.

### Tissue doppler imaging (TDI)

TDI is an approach that uses the Doppler technique to examine the velocity of myocardial tissue motion during the cardiac cycle [42]. It helps evaluate the mitral annular motion velocity. The E/E’ ratio, which compares early transmitral velocity to TDI mitral annular early diastolic velocity, offers an indicator of diastolic dysfunction when the value exceeds 15 mmHg. A decreased ratio of early ventricular filling to atrial-driven ventricular filling (E/A ratio) signifies diastolic impairment. The TDI also helps measure strain and strain rates at the global and regional levels in different myocardial segments. It provides a direct estimate of intrinsic myocardial contractility. The quantification of fibrosis is also possible with TDI by measuring longitudinal, circumferential, and radial contractions in myocardial fibres. These procedures help to elicit early DbCM among asymptomatic T2DM patients [19].

The E/E’ ratio, though widely used as a diastolic function indicator, shows variable diagnostic performance across different populations and disease states, with some studies reporting lower sensitivity in certain patient subgroups, particularly those with preserved ejection fraction [43].

### Multidimensional echocardiography

Three-dimensional echocardiography (3D) involves the collection of pyramidal (volume-based) datasets in real time rather than triangular data or sector-shaped slices in conventional systems. It can identify ventricles with abnormal shape and mobility, along with the routine assessment of dysfunction [44]. Four-dimensional (4D) echocardiography is 3D echocardiography with temporal resolution. Here, tomographic images (cross-sectional views of internal structures reconstructed from imaging data) are used to detect strain and strain rates more accurately and reduce interference [45].

The characteristics that may be specifically noted in DbCM are the ejection fraction, strain rate, and the hypertrophy status [46]. Advanced echocardiographic techniques can detect subtle changes like slightly lower LVEF, suggesting early systolic impairment. Similarly, concentric adverse remodelling and LV hypertrophy can be detected even in asymptomatic patients [47].

The 3D and 4D echocardiography represent promising advances in comprehensive cardiac assessment, however their clinical implementation faces certain technical limitations, including inferior spatial and temporal resolution compared to 2D methods, operator dependency requiring specialized training, and reduced feasibility (63–83%) due to acoustic window requirements and patient cooperation needs [48]. The 4D strain analysis shows more potential for myocardial assessment, but further validation studies are needed to establish standardized reference values and improve inter-vendor reproducibility before it can be routinely implemented in diabetic cardiomyopathy screening protocols.

### Magnetic resonance imaging (MRI)

MRI is the benchmark test for assessing left ventricular volume, function, or mass. It evaluates the cardiac chamber size, left ventricular ejection fraction, cardiac mass distribution, and tissue characteristics [49]. It offers excellent spatial and temporal resolution. This technique helps to quantify triglycerides and detect biventricular changes and the extent of fibrosis. Phase magnetic resonance imaging (P-MRI), a specialised form of MRI, allows measurement of blood flow and tissue motion. It helps in the diagnosis of many other prognostic parameters, such as the mitral valve inflow velocity, early deceleration time, pulmonary vein flow velocity, intramyocardial velocity, and hypertrophy. Phase-contrast MRI, though promising for detailed flow assessment, demonstrates reproducibility concerns, particularly for low-velocity measurements (< 6.4 cm/s), with accuracy errors of up to 276% for peak velocities in the lower ranges typical of cardiac applications, potentially limiting its precision in diabetic cardiomyopathy screening [50]. Despite these implementation barriers, ongoing technological advances and cost-effectiveness demonstrations continue to support MRI’s expanding role in comprehensive cardiac tissue characterization and metabolic assessment.

### Novel techniques for diagnosis

Two- and three-dimensional speckle tracking echocardiography (2D/3D-STE) is a relatively recent imaging technique that enables the quantification of cardiac mechanical deformation (strain and strain rate) by tracking myocardial motion throughout the cardiac cycle. First, it analyses the sequence of images from two-/three-dimensional echocardiography. Then, it analyses the distance between pixels during the cardiac cycle. Reduced noise and interference provide a more accurate diagnosis of cardiac dysfunction at a very early stage of diabetes [51, 52]. Zimmermann et al. (2024) reported that speckle-tracking analysis after peak exercise in type 1 diabetes revealed subtle impairments in myocardial strain and diastolic recovery, highlighting the value of stress echocardiography in detecting early, subclinical dysfunction relevant to DbCM [53]. Further advancements in spatial and temporal resolution are needed to enhance the diagnostic accuracy and clinical utility of imaging modalities.

Molecular MRI is another advanced technology that can pinpoint the free radical level, source, or tissue type. It involves labelling extracellular surface receptors with targeted contrast agents and detecting them with an antibody-tagged probe. Since free radicals and oxidative stress play vital roles in the pathogenesis of DbCM, their abnormal presence can help in the diagnosis and prognosis of DbCM [54]. Delayed gadolinium-enhanced cardiac MRI is frequently used for the non-invasive measurement of focal collagen deposits or replacement fibrosis that exemplifies scar formation resulting from myocardial injury or infarction [55]. Since diffuse collagen deposits characterize DbCM, measuring extracellular volume is more precise on the basis of myocardial and blood T1 mapping information preceding and following the administration of contrasting agents and the patient’s haematocrit value [56, 57].

Magnetic resonance spectroscopy (MRS) is a fast-developing, non-invasive, non-radiation-based technique that clearly shows the metabolic pathways within cells. The H^1^ MRS, P^31^ MRS, and the latest C^13^ MRS are useful for detecting and quantifying specific cardiac biomolecules. The H^1^ MRS and C^13^ MRS help detect carbohydrate and lipid metabolites. P^31^ MRS helps determine the phosphocreatine and ATP amounts (the ratio of which decreases in DbCM). MRS examines the triglyceride content to identify the extent of steatosis. MRS can also predict clinical HF, making it an up-and-coming technique in this scenario [58]. 

### Biomarkers of DbCM

Conventional cardiac biomarkers, such as atrial natriuretic peptide and N-terminal proBNP (NT-proBNP), can predict diastolic dysfunction and HF. NT-proBNP has been extensively used in major heart failure trials, including DAPA-HF [59]. It has now emerged as the most extensively studied biomarker for DbCM, with robust evidence supporting its diagnostic and prognostic utility in diabetic populations [60, 61]. Comparative cohort studies and randomised trials revealed that it plays a pivotal role in the comprehensive evaluation of diabetic patients with heart failure, facilitating appropriate management strategies and predicting hospitalization risk. The NT-proBNP is thus an insightful biomarker for assessing cardiac health beyond conventional imaging assessments in diabetic populations [62]. However, additional studies are needed for their application in screening asymptomatic individuals for characteristic features of DbCM [63].

Troponin, a complex with three regulatory proteins (TnT, TnI, and TnC) involved in cardiac and skeletal muscle contraction, is a putative marker of cardiac injury or infarction due to its sensitivity and specificity. Phosphorylated troponin levels are elevated in diabetes mellitus patients, but their correlation with DbCM has yet to be independently proven. High-sensitivity cardiac troponin T is often associated with adverse diastolic dysfunction but not systolic dysfunction, revealing its potential as an early biomarker of subclinical manifestations of ventricular dysfunction [64]. 

A recent study revealed an explosion of biomarkers for screening, diagnosis, or therapeutic response. Cardiotrophin-1, a biomolecule of the glycoprotein 130 (gp130) family, is considered an inducer of cardiomyocyte hypertrophy. It is a promising candidate because glucose, insulin, angiotensin II, reactive oxygen species (ROS), hypoxia, and mechanical stretch stimulate it. It modulates myocardial contractility, cardiac conduction, fibrosis, cardiac remodelling, and glucose metabolism. Being proven to be correlated with left ventricular hypertrophy, making it an excellent marker for diagnosing cardiac dysfunction in its early stages [65, 66].

The study by Shaver et al. (2016) investigated serum biomarkers for the early detection of DbCM in a West Virginian population. Their analysis on this highly diabetic prevalent population validated a panel of biomarkers, such as the profibrotic marker TGF-β, and the insulin resistance marker IFGBP7, for the early diagnosis of DbCM [67]. IGFBP-7 demonstrates strong independent prognostic value as a reliable biomarker for heart failure patients across the spectrum of ejection fraction, including both HFrEF and HFpEF populations [68].

Matrix metalloproteinases (MMPs) and tissue inhibitors of metalloproteinases (TIMPs) form the proteolytic system that regulates extracellular matrix (ECM) homeostasis [69]. The level of MMPs is correlated with cardiac remodelling and LVDD in HF patients [70]. The MMP/TIMP ratio may also reflect the progression of LV remodelling in patients [71]. An enzyme‑linked immunosorbent assay (ELISA) may be used to analyse the levels of MMPs to diagnose the development of DbCM [72]. Elevated serum levels of PIIINP, along with Galectin-3 and ST2, have been shown to correlate with impaired left ventricular diastolic function, suggesting their potential as non-invasive surrogates for structural and functional myocardial abnormalities, including myocardial fibrosis as assessed by late gadolinium-enhanced cardiac MRI [73].

Many recent studies have determined their role as biomarkers of cardiac dysfunction, hypertrophy, and fibrosis. MicroRNAs are small noncoding RNAs that are involved in the posttranscriptional regulation of more than one-third of the genes that are expressed [74]. Recent investigations demonstrate that miR-223-3p modulates the NLRP3 signaling pathway to alleviate myocardial inflammation and pyroptosis, while miR-34a-5p influences cardiotoxicity through the Sirt1/p66shc axis, and miR-214-3p participates in cardiac fibrosis regulation via LncRNA-MIAT interactions. Notably, comprehensive multiomics analysis by Balakrishnan et al. (2025) identified hsa-miR-302a-3p as the critical miRNA regulating AKT1, the highest-ranked hub gene in DbCM, while other studies confirm its broader therapeutic potential by inhibiting epithelial-mesenchymal transition in diabetic complications and providing cardioprotective effects against reperfusion injury. Though these findings underscore the substantial clinical potential of miRNAs as biomarkers, there is a critical need for expanded research to fully elucidate their diagnostic utility and therapeutic applications [2, 75].

DbCM, diabetes, or any metabolic syndrome, are characterized by persistent low-level inflammation and increased levels of oxidative stress. Metabolic and inflammatory factors trigger the typical inflammatory biomarker C-reactive protein (CRP), which is produced by the liver and regulated by adipocyte-derived proinflammatory cytokines such as interleukin-6 and tumor necrosis factor (TNFα). Studies have also shown the significance of biomarkers of chronic inflammation for the development of therapeutic, diagnostic, or prognostic examinations [76].

While several experimental biomarkers have been proposed for DbCM, only a limited subset has undergone stronger validation in clinical studies. Natriuretic peptides (BNP, NT-proBNP) remain the most widely validated and clinically used biomarkers, with prognostic significance for heart failure hospitalization and mortality in diabetic patients [62]. High-sensitivity cardiac troponins (hs-cTnT, hs-cTnI) are also well validated for detecting subclinical myocardial injury in diabetes and predicting progression to overt DbCM [64]. In contrast, markers such as IGFBP-7, and fibrosis-related proteins are promising but remain in earlier validation phases, while microRNAs and other experimental markers are still investigational and in the preclinical research stage.

At present, no universally accepted diagnostic criteria for DbCM exist, which complicates both clinical recognition and research comparability. Based on current evidence, a pragmatic framework may be envisioned rather than formalized. Such an approach could begin with the clinical context of longstanding diabetes in the absence of alternative causes of cardiomyopathy, supported by imaging findings suggestive of early diastolic dysfunction or subtle systolic impairment on echocardiography or cardiac MRI. These observations may be further strengthened by biomarker evidence (e.g., natriuretic peptides, high-sensitivity troponins, or emerging candidates such as cardiotrophin-1 and HFABP). Finally, the exclusion of other structural or ischemic heart disease remains central to ensuring diagnostic specificity.

DbCM can be differentiated from ischemic cardiomyopathy (ICM) by its pattern of dysfunction and imaging features. While ICM typically presents with regional wall motion abnormalities corresponding to coronary territories, DbCM is characterized by global, symmetric impairment [77, 78]. Some studies in T2DM without known ischemic heart disease report non-ischemic, mid-myocardial LGE lesions (often in basal lateral or inferolateral segments), preserved regional motion in those areas, and associated fibrosis and diastolic impairment. This is different from the pattern for ischemic cardiomyopathy. Thus, LGE pattern analysis (distribution, transmurality, and segmental involvement) can help differentiate DbCM from ischemic etiologies in clinical practice [79, 80]. DbCM must also be separated from infiltrative cardiomyopathies such as amyloidosis or sarcoidosis. In DbCM, histology typically reveals interstitial fibrosis, cardiomyocyte hypertrophy, and microvascular changes, but lacks the characteristic infiltrative deposits of amyloidosis or granulomas of sarcoidosis. Early in its course, DbCM tends to preserve normal or only mildly increased wall thickness, whereas infiltrative cardiomyopathies often cause marked thickening. Moreover, DbCM does not exhibit extracardiac manifestations, which are common in systemic infiltrative diseases [81]. While not intended as definitive criteria, this integrative perspective can serve as a conceptual scaffold, encouraging future work toward standardized, consensus-driven definitions of DbCM.

To integrate these assessments into clinical practice, Table 2 summarizes the key imaging modalities, biomarker assays, and invasive techniques required for comprehensive evaluation of myocardial fibrosis, remodeling, and diastolic dysfunction. Given the multifactorial nature of the disease, a multimodal diagnostic approach is often recommended for the accurate detection of DbCM. Although no specific biomarkers for DbCM are currently established in routine clinical practice, a combination of molecular markers and imaging modalities shows promise for improving diagnosis. Timely diagnosis and treatment of DbCM can help slow the progression of the disease and improve patient prognosis.

**Table 2:** Assays for assessing myocardial fibrosis, remodeling, and diastolic dysfunction

Assay type	Method/Marker	Clinical relevance	Reference
Circulating biomarkers	NT-proBNP, ANP, troponins, cardiotrophin-1, TGF-β, IGFBP7, MMPs, CRP, microRNAs	Reflect hemodynamic stress, cardiomyocyte injury, fibrosis, and remodeling; some (e.g., NT-proBNP, troponins) are validated in HF and applied in DbCM studies	[62, 64, 65, 68]
Echocardiography	Tissue Doppler Imaging (TDI), speckle-tracking strain (longitudinal, circumferential, radial)	Detects early diastolic dysfunction, strain abnormalities, and subtle remodeling before overt HF	[19, 51]
Cardiac MRI	Late gadolinium enhancement (LGE), T1 mapping, extracellular volume (ECV) quantification	Gold standard for detecting focal and diffuse myocardial fibrosis, ventricular remodeling, and prognosis	[49]
Endomyocardial biopsy	Collagen volume fraction, collagen I/III ratio, and TGF-β1 expression	Directly quantity fibrosis and molecular markers	[81]

## Prevention of DbCM

Regular exercise is essential for preventing any lifestyle disorder or related complications [82]. The intensity of training can be determined on the basis of age, physical conditions, and other morbidities. Exercise improves blood circulation and homeostasis. An increase in blood flow improves the delivery of insulin and glucose to cells [83], increasing their uptake and possibly reducing hyperglycemia. Exercise-induced hemodynamic stress leads to adaptive remodelling of the heart, which enhances its contractile function [84]. Research has shown that exercise-trained individuals exhibit enhanced systolic and diastolic function [85]. A low-carbohydrate and low-fat diet is strictly recommended for diabetic patients to prevent progression to cardiomyopathy and HF. A healthy diet reduces glucotoxicity and lipotoxicity. A diet rich in vegetables, fruits, and whole grains offers a distinctive combination of antioxidants, vitamins, minerals, and phytochemicals. Resistant starches that are digested very slowly, mainly by fermentation in the large intestine, are highly recommended. They reduce serum glucose and cholesterol levels and prevent fat accumulation [86]. In addition, they contain phytochemicals such as phenolics, lignans, carotenoids, β-glucans, and inulin, and a fibre-rich diet results in decreased blood glucose excursions and attenuated insulin responses [16].

Regular monitoring of glucose levels is also essential to prevent hyperglycemia. Continuous glucose monitoring (CGM) devices attached to the body provide real-time data on glucose levels. The insulin‑only configuration of a bionic pancreas, receiving CGM data and initialized only via body weight, autonomously controls basal, correction, and meal boluses without carbohydrate counting. This system has demonstrated superior time‑in‑range, reduced HbA1c, and improved glycemic outcomes compared to standard insulin therapy in type 1 diabetes [87].

Drugs such as antidiabetic medications, metformin, and insulin supplementation help lower glucose levels, which will be discussed in the following section.

## Management of DbCM

In addition to lifestyle modification, interventions for DbCM include therapeutic control of blood glucose and insulin levels, reducing injury to cardiomyocytes, reducing hypertrophy and fibrosis, addressing hay-wired signalling pathways, and delaying HF.

### Antidiabetic medications

Diabetes is the major risk factor for DbCM, and antidiabetic medications constitute the current benchmark standard intervention mechanism for DbCM. Importantly, beyond glycemic control, several of these agents exert direct cardiovascular benefits, improving outcomes in heart failure and reducing mortality, as shown in large outcome trials such as EMPA-REG OUTCOME [88], CANVAS [89], DECLARE-TIMI 58 [90], and LEADER [91]. Metformin remains the primary treatment option for T2DM [92], although its evidence for direct cardioprotective effects in DbCM is less consistent compared to newer drug classes. Glucagon-like peptide modulators or sodium‒glucose cotransporter 2 inhibitors constitute the second line of therapy for stimulating insulin release and facilitating glucose uptake into cells [93], with both classes now recognized for their cardiovascular benefits beyond glucose lowering, including reduction in heart failure risk and adverse cardiac remodeling. Insulin is often the third line of treatment unless the patient is critically ill or during surgeries and other emergencies when hyperglycemia is uncontrollable by other antidiabetic drugs [15, 94]. The various management strategies, mechanisms, effects, and challenges are listed below (Table 3).

#### Glucagon-like peptide-1 (GLP-1) receptor agonist

GLP1 is a peptide hormone secreted by the epithelial L cells of the intestine in response to meal intake. It is also called incretin hormone because it triggers glucose-stimulated insulin release and reduces glucagon secretion. This helps to suppress postprandial glucose excursions in the blood [95]. GLP-1 receptor agonists have also been proven to reduce fibrosis, inflammation, and oxidative stress in diabetic cardiomyopathy models, demonstrating their role in cardioprotection [96]. GLP-1 agonists improve the LV ejection fraction, myocardial contractility, endothelial function, coronary blood flow, and output [97]. Lixisenatide and liraglutide are classic examples.

**Table 3:** Management of diabetic cardiomyopathy

Management	Drugs	Mechanism	Effects	Challenges	References
GLP-1 receptor agonists	Liraglutide	Stimulates the glucagon-like peptide-1 receptor to enhance insulin release	Increases Myocardial Glucose Oxidation Rates, Lower glycated hemoglobin levels without causing side effects like weight gain or hypoglycemia, Cardioprotective effects	Slight gastrointestinal side effects	[98]
DPP4 inhibitors	Evogliptin	Reduces blood glucose and HbA1c levels	reduces lipotoxicity and mitochondrial injury	Arthralgia	[99]
SGLT2 inhibitors	empagliflozin	Glucose-lowering and natriuretic action	Enhance myocardial metabolism, restore mitochondrial function, and address microcirculatory issues. Reduce myocardial fibrosis, decrease oxidative stress, prevent programmed cell death, and regulate autophagy, inhibiting ER stress, and regulating the intestinal flora	Reduced blood pressure, hypovolemia urinary and genital tract infections, kidney injury, fracture, amputation, ketoacidosis	[100]
Anti-hyperglycemic agent	Metformin	activate the PK2/PKR-mediated AKT/GSK3β pathway	Precludes any glucose and lipid metabolism dysfunction, cardiomyocyte apoptosis, fibrosis, and cardiac insufficiency	gastrointestinal side effects, vitamin B12 deficiency	[101, 102]
Anabolic peptide hormone	Insulin	Reduces hepatic gluconeogenesis and reduces triglyceride production Prevents lipolysis in adipose tissue and proteolysis in the whole body and muscles while promoting glucose uptake in muscle	Manages hyperglycemia	Sodium and fluid retention, especially in patients with heart failure, is risky	[103, 104]

GLP-1, glucagon-like peptide 1; DPP4, dipeptidyl peptidase 4; SGLT2, sodium‒glucose cotransporter 2; PK2, prokineticin 2; PKR, protein kinase R; AKT, Ak strain transforming; GSK3β, glycogen synthase kinase-3 beta

#### Dipeptidyl peptidase 4 (DPP4) inhibitors

DPP4 inhibitors, oral antidiabetic drugs approved by the FDA as early as 2006, have provided promising results for hyperglycemia [105]. DPP4, a ubiquitous protein, exhibits exopeptidase activity against GLP-1. DPP4 inhibitors provide a physiological increase in GLP-1 levels. In addition to affecting glucose metabolism, it regulates inflammation, cell survival, and vascular function, lowering HbA1c levels [106]. It reduces the threat of hypoglycemia, and the possibility of weight gain is not present. However, an increase in upper respiratory tract infections, hypertension, a reduction in T lymphocytes, pancreatitis, and hepatic enzyme elevation are noted. The SAVOR-TIMI 53 trial also revealed a slight risk of HF with saxagliptin. However, the EXAMINE, TECOS, and VIVIDD trials have not demonstrated such dangers for the cardiac system with DPP4 inhibitors. Combination therapy with other drugs might prove beneficial. β-blockers (β-adrenergic blocking agents) have shown great promise in attenuating HF risk. Examples of DPP4 inhibitors are sitagliptin, saxagliptin, linagliptin, and alogliptin [107].

#### Sodium‒glucose cotransporter 2 (SGLT2) inhibitors

SGLT-2 inhibitors decrease renal tubular glucose reabsorption, reducing blood glucose levels without stimulating insulin release. These drugs work via the excretion of glucose along with sodium through the urine. SGLT2 is located within the kidney’s proximal tubule, where 90% of glucose reabsorption occurs from the glomerular filtrate. The EMP-REG and EMPRISE trials showed that empagliflozin lowers the risk of cardiovascular death and hospitalization related to HF. In addition to its glucose-lowering effects, it has cardioprotective effects, making it a better candidate for treating DbCM. It plays significant roles in cardiac iron homeostasis, mitochondrial functions, and the renin‒angiotensin‒aldosterone system (RAAS) pathways. It also has anti-inflammatory, antifibrotic, and antioxidant effects on the cardiac system [108]. The limitations include urinary tract infections, probably due to glucosuria. The FDA has also recommended monitoring renal function and blood volume status as precautionary measures to avoid adverse effects on the kidneys [109]. SGLT2 inhibitors can also be used along with GLP antagonists to reduce the risk of cardiac diseases and diabetes [108, 110]. Although the absolute benefits seem to be greatest for those with established cardiovascular disease, the CANVAS study (CANagliflozin cardiovascular assessment) has shown that this class of medications significantly lowers adverse cardiovascular events, including HF, among people with diabetes [111]. Human myocardial studies have shown that SGLT2 inhibitors reduce JunD expression, a transcription factor implicated in oxidative stress and adverse remodeling, thereby improving contractile function [112].

Large-scale randomized cardiovascular outcome trials such as SAVOR-TIMI 53 (*n* ≈ 16,492), EXAMINE (≈ 5,400), TECOS (~ 14,700), and the VIVIDD trial (~ 250), offer robust internal validity in high-risk diabetic populations, though their relatively short to medium durations and selective inclusion criteria limit generalizability to broader or lower-risk T2DM cohorts. Meanwhile, SGLT-2 inhibitor studies, including EMPA-REG OUTCOME (*n* ≈ 7,028) and the CANVAS Program (*n* ≈ 10,000), consistently reduced heart failure hospitalization and cardiovascular mortality, reinforcing their clinical impact. Real-world evidence from EMPRISE—with over 16,000 patients per arm in interim analyses, enhances external validity, though being observational, it remains vulnerable to residual confounding. Overall, these trials demonstrate considerable strengths in design and sample size, yet highlight the need for longer-term, diverse-population studies to ensure broader applications.

#### Metformin

Metformin has been the first-line treatment for diabetes and related comorbidities for the last three decades. It belongs to the biguanide family of drugs, which reduces hepatic gluconeogenesis and improves insulin sensitivity. The molecular mechanism of action may include the activation of AMP-activated protein kinase, which decreases ATP expenditure pathways and stimulates ATP production pathways such as glucose uptake by tissues and glucose and fatty acid oxidation, reducing ROS production and improving insulin resistance. Metformin thus reduces cardiac remodelling, interstitial fibrosis, hypertrophy, and diastolic dysfunction [113]. Lactic acidosis, gastrointestinal side effects, or B12 deficiency rarely occur, and renal function must be monitored regularly [114].

#### Insulin

Although insulin was discovered to treat diabetes, it is not used as a first-line therapy for hyperglycemia unless β cells of the pancreas are not produced enough to meet the body’s requirements, as in type 1 diabetes mellitus. However, it is famous as the third line of therapy for maintaining hyperglycemia and glucotoxicity [115]. It is preferably considered in combination with antihyperglycemic agents, especially when the HbA1c level is ≥ 10% (≥ 86 mmol/mol) [94].

#### Emerging experimental therapies

Beyond established glucose-lowering agents, experimental studies have explored adjunctive pharmacological strategies. For instance, Tappia et al. (2024) demonstrated that 5-HT2A receptor blockade with sarpogrelate in chronic diabetic models significantly improved ventricular contractility and reversed subcellular defects, restoring Ca2+-ATPase activity, reducing mitochondrial swelling, and preserving myofibrillar architecture, highlighting a novel cardioprotective strategy in DbCM [116].

Recent translational research by Fiordelisi et al. (2024) demonstrated that L-Arginine supplementation represents a promising mitochondrial therapy for DbCM, effectively preserving diastolic function and exercise tolerance through restoration of cardiac mitochondrial fitness [117]. Hashiesh et al. (2024) found that activation of the CB2 receptor with β-caryophyllene markedly attenuated AGE/RAGE-induced oxidative stress, suppressed TGF-β/Smad-driven fibrosis, enhanced the PI3K/AKT/Nrf2β antioxidant pathway, and inhibited NLRP3 inflammasome activation in diabetic cardiomyopathy [118].

Rostami et al. (2024) demonstrated that activation of PPARβ/δ plays a protective role in DbCM by suppressing inflammation and fibrosis, key drivers of myocardial remodeling. The protective effects of PPARβ/δ activation appear to stem from its ability to suppress MAPK signaling, leading to reduced NF-κB and AP-1 transcriptional activity. Consequently, PPARβ/δ agonists may hold therapeutic potential in limiting inflammation and fibrosis during the course of DbCM [119].

### Biomarkers of drug response

The heterogeneity in glucose-lowering drug responses among patients depends on genetic and environmental factors. However, HbA1c is widely accepted by regulatory bodies, such as the FDA, as a biomarker and predictor of efficacy in clinical trials [120]. The sitagliptin study has elucidated specific target engagement as another excellent biomarker for drug response [121]. Differential values of metabolites such as citric acid, myoinositol, and hippuric acid were used to predict metformin responses in a study conducted in Korea [122].

### Resistance to treatment

Treatment resistance represents a critical challenge in DbCM management, where therapies effective in early diabetes stages lose their efficacy in advanced disease. While hyperglycemia-targeting treatments successfully lower HbA1c levels in early diabetes, their cardiovascular protective role diminishes significantly in later stages, particularly regarding heart failure prevention and mortality reduction [123]. This phenomenon was strikingly demonstrated in the Action to Control Cardiovascular Risk in Diabetes (ACCORD) trial, where intensive therapy achieved HbA1c levels of 6%, yet paradoxically increased mortality risk by 22% [124]. The Action in Diabetes and Vascular Disease: Preterax and Diamicron Modified Release Controlled Evaluation (ADVANCE) trial [125], targeting HbA1c of 6.5%, similarly revealed concerning mortality trends despite stringent glycemic control, indicating treatment resistance to conventional glucose-lowering approaches. Furthermore, dedicated heart failure trials including Examination of Cardiovascular Outcomes with Alogliptin versus Standard of Care (EXAMINE) trial [126] and Trial Evaluating Cardiovascular Outcomes with Sitagliptin (TECOS) trial [127] showed no significant reduction in heart failure hospitalizations, while the Functional Impact of GLP-1 for Heart Failure Treatment (FIGHT) study demonstrated that liraglutide had no impact on mortality or hospitalizations in established heart failure patients [128]. These findings collectively illustrate that once traditional antidiabetic therapies exhibit treatment resistance, novel therapeutic approaches beyond glucose control will be necessary.

#### Hypoglycemia

A stringent standard of care to control the glycemic index could lead to hypoglycemia. Below a certain point, hypoglycemia is a serious concern. It can also affect overall health conditions, especially cardiac and cerebral health. Hypoglycemia triggers the autonomic system, inducing an avalanche of actions, including the release of epinephrine, stimulating glucagon secretion, enhancing blood flow to the brain to prevent neuroglycopenia, and promoting gluconeogenesis in the liver to restore blood glucose levels. Acute situations can lead to convulsions, coma, or sudden cardiac death [129]. The problem can be managed with intravenous dextrose, followed by an infusion of glucose, or glucagon can be administered if there is no intravenous access [130]. If the patient is conscious, oral medications or easily absorbable carbohydrate sources can be given. Once the patient is completely awake and aware, more complex carbohydrates are given, along with regular glucose level monitoring to maintain euglycemia. Patient education about the ramifications of frequent hypoglycemic attacks and their consequences is advised. Patients should also be encouraged to wear medical alert bracelets or necklaces and carry sugar candies or tablets in emergencies [131].

#### Heart failure (HF)

Undiagnosed and untreated DbCM usually culminates in HF. During the initial stages of HF, patients are immediately hospitalized. Oxygen levels are monitored directly (PaO2 < 60% or SaO2 < 90%) [132]. Non-invasive positive pressure ventilation (NIPPV) is administered in instances of respiratory distress [133]. Depending on the symptoms, the following pharmacological drugs are administered. Diuretics (thiazides, loop diuretics, and potassium-sparing) and salt restriction (to reduce fluid retention) are advised for patients with HF symptoms and a low left ventricular ejection fraction (LVEF) to ease their symptoms [134]. ACE inhibitors or angiotensin receptor blockers (ARBs) are used for neurohormonal modulation, vasodilation, and improvement in LVEF (in patients who are not sensitive to ACE inhibitors and ARBs, hydralazine and nitrates are used as substitutes). Beta-adrenergic blockers alter neurohormonal processes, increase symptoms and LVEF, increase survival, prevent arrhythmias, and regulate the ventricular pace. Aldosterone antagonists, which are used with other medications, can improve heart rate variability, reduce ventricular arrhythmias, and reduce the burden on the heart [135].

## Quality of life of DbCM patients

From the onset of prediabetes to the chronic stages of DbCM and HF, the health conditions of individuals decrease. The quality of life is very much compromised. It is problematic and poses high challenges for caretakers and patients. DbCM is most frequently underdiagnosed, and therapy is not well defined. Without proper awareness of complications, which ultimately lead to HF, most patients lack motivation for self-care or lifestyle changes. The adverse effects of certain medications also reduce adherence to therapy. Quality of life is adversely affected as patients become comorbid. In such cases, when the disease progresses, the cost also becomes a hindrance to compliance with treatment, leaving most of the aged patients to their fate. This scenario is not limited to just an economic burden, a social burden for society, or a psychological burden for patients and their loved ones.

Health-related quality of life (HRQoL) is a concept developed to investigate the impact of health status on quality of life [136]. The various factors that affect the HRQoL score are listed in the figure below (Fig. 2). Another database used to assess quality of life is the PROQOLID (Patient-reported Outcome and Quality of Life Instruments database), which has over 900 instruments, the majority of which are surveys or questionnaires for measuring patient-centered outcomes [137, 138].

**Fig. 1:**
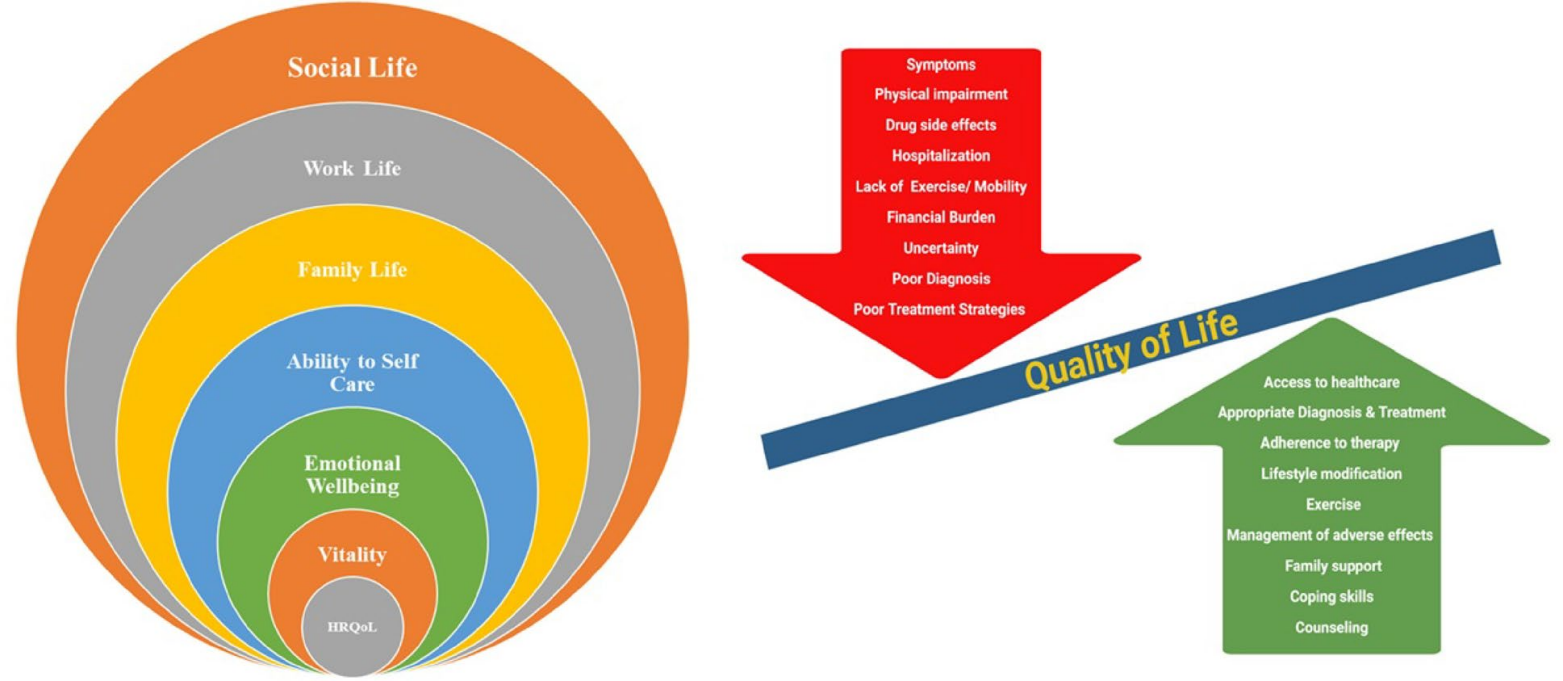
Factors affecting the quality of life of DbCM patients. (**A**) Quality of life, which is impacted by disease and treatment, is often referred to as HRQoL (health-related quality of life). HRQoL is affected by the energy/viability of the patient, emotional well-being, ability to self-care or even care for others, how well they can lead a family life and work life, or how well they can contribute to society. (**B**) Quality of life is influenced by many factors, as listed above

Big data generated from hospitals and other electronic health records, social media databases, or patient monitoring devices is also a significant source for analysing and predicting trends in patient disease progression and quality of life [139]. Mild exercise increases blood circulation and improves overall health in any disease condition [140].Proper diet control [86] and the use of required medications can reduce risks severalfold, and individuals can lead an independent life. However, a lack of awareness to address these issues can worsen or complicate the scenario.

## Outcomes and natural history of DbCM

DbCM carries a significant clinical impact. Data from cohorts and large diabetes trials indicate higher risks of heart failure and mortality, but its trajectory is less clearly defined than that of other cardiomyopathies. Table 4 outlines major studies on DbCM outcomes, including natural history cohorts.

**Table 4:** Outcomes of DbCM

Study (first author, yr)	Design & population	N, time period	Main outcomes	Key limitations	Reference
Dandamudi 2014	Cross-sectional survey on randomly selected residents of age above 45years in Minnesota	2042 subjects; 3years	Community prevalence rate is 1.1%, mortality among DbCM was 18% and HF was 22% during follow-up	Variable DbCM definitions; older cohort design	[141]
Sani 2022	Longitudinal population based-study of adults above 18years of age in Malaysia	1355 subjects; 15-years	Higher mortality rate in the diabetic cohort; supports adverse prognosis	Single-cohort, limited sample, residual confounding	[142]
Medhekar 2020	Cardiomyopathy patients from a single centre, subgroup analyses	18,003 subjects; 3.35 years	Diabetic status predicts more deaths & hospitalizations in HFrEF	Retrospective single-centre design; possible comorbidity confounding	[143]
Kong 2020	Prospective cohort study of subjects enrolled in Korean Acute Heart Failure Registry	5625 subjects; 8 years	4.4% in-hospital mortalities and 46.3% overall mortalities. Diabetes and HbA1c ≥ 7.0% were significantly associated with mortalities by HFrEF	It is an observational cohort design and may introduce residual confounding	[144]

DbCM most often evolves from a prolonged subclinical stage that is often characterized by metabolic myocardial remodeling, interstitial fibrosis, and early diastolic dysfunction into symptomatic heart-failure over months to years. Progression rates vary by cohort, but observational studies and registry data indicate that a meaningful proportion of individuals with subclinical abnormalities later develop clinical HF and experience increased medium- to long-term mortality compared with non-diabetic populations [142, 144]. Modifiers of this trajectory include glycemic control, obesity, hypertension, ischemic heart disease, and access to guideline-directed therapies.

When adjusted for standard measures of cardiac dysfunction, patients with diabetes generally show worse outcomes-higher rates of HF hospitalization and mortality—than non-diabetic patients with similar degrees of systolic impairment, as reported across cohort and pooled analyses [143]. Randomized CVOTs in diabetes support a consistent reduction in HF events with SGLT2 inhibitors across risk strata, reinforcing the concept that diabetes both increases HF risk and offers a therapeutic target that modifies prognosis; however, many RCT populations were enriched for established cardiovascular disease, and observational data are subject to confounding, so absolute generalizability remains constrained [88, 111, 145]. Overall, DbCM is both prevalent and prognostically adverse, underscoring the need for standard diagnostic criteria and prospective studies, which is discussed in the following section on *Future Directions.*

## Future directions

**Fig. 2:**
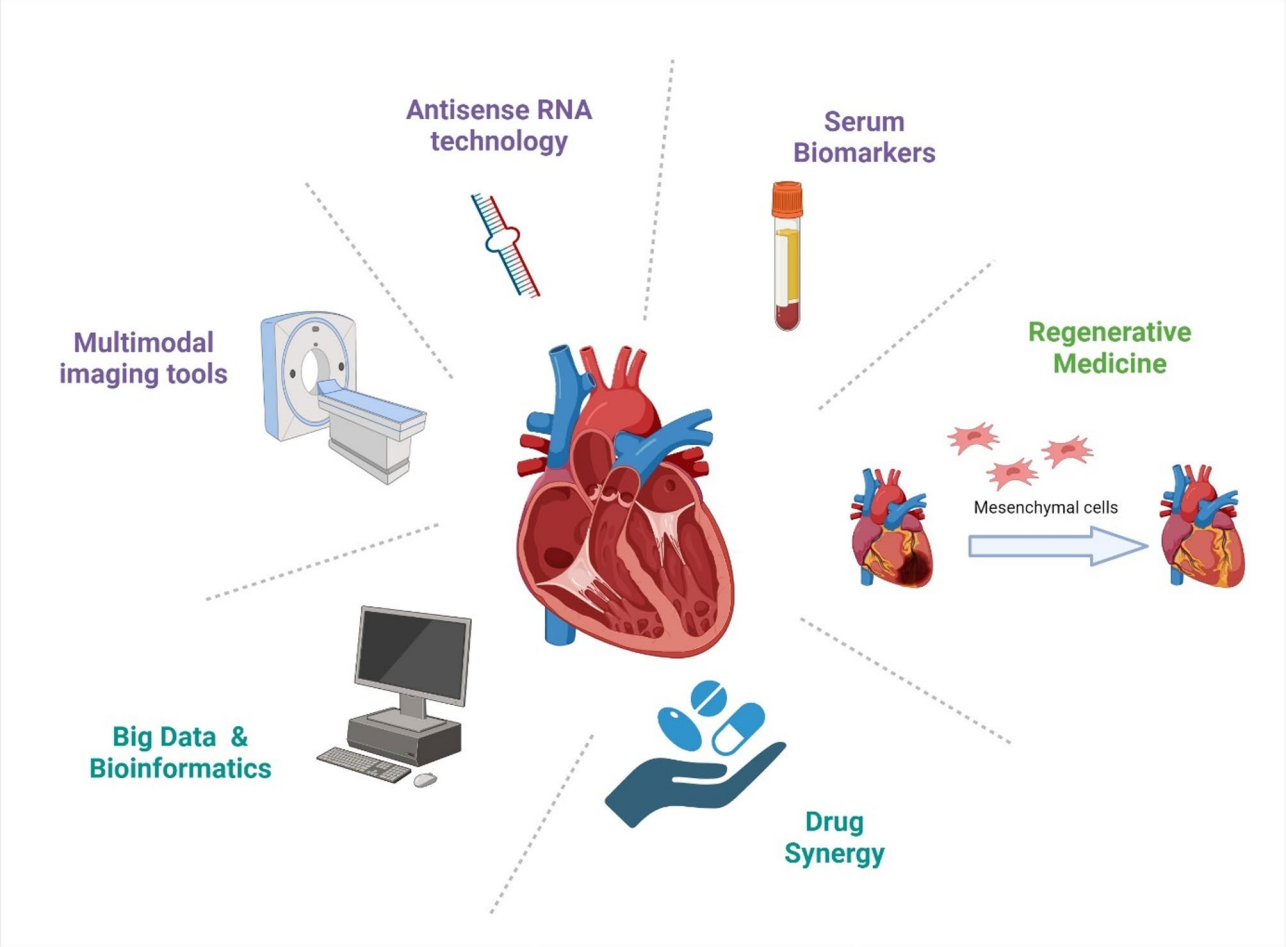
Schematic representation of different approaches to diagnose and treat diabetic cardiomyopathy

The rising prevalence of youth-onset diabetes (< 40 years) is expected to markedly increase the burden of DbCM in the coming decades. Evidence shows that younger individuals with diabetes experience earlier onset of heart failure and worse long-term outcomes compared with later-onset or non-diabetic populations. This underscores the need for early detection and targeted interventions in younger cohorts [146, 147]. Research to develop better diagnostic and therapeutic techniques to detect DbCM and address pathological progression at early stages is crucial. This will also help better characterize the disease and its stages [148–150]. Development in the field of imaging modalities will help better understand patient prognosis. The various approaches followed to treat and diagnose DbCM are represented in this section (Fig. 2).

### Improved diagnostic tools

Multimodal imaging tools are very promising technologies in the field of cardiac radiology. It helps simultaneously produce optical, magnetic, or radioactive signals for many techniques, such as CT, MRI, SPECT (single-photon emission computed tomography), or PET, enhancing early diagnosis and prognosis [151]. The fusion of images from different modalities also has excellent potential for obtaining three-dimensional composite images with greater detail [152]. Large randomized clinical trials in early-stage diabetic cohorts involving the use of multiple imaging modalities to understand the characteristics of the disease for early diagnosis in the future and the design of effective treatment protocols are needed. Cross-trained cardiac imagers in multiple modalities are also needed.

### Serum biomarkers

There is a dire need to develop predictive biomarkers of DbCM in blood samples. Minimally invasive and significantly less time-consuming tests are the most encouraging for patients. Biomarkers can also be designed for prognosis, treatment response, drug response, or risk assessment. Biomolecules associated with cardiac hypertrophy, myocardial injury, mitochondrial damage, oxidative stress, glucotoxicity, lipotoxicity, inflammation, and fibrosis are usually screened for this purpose. A recent study identified a heart-specific biomarker, heart-type fatty acid binding protein (hFABP), associated with prediabetes and showed encouraging results as an indicator of coronary disease [139]. Most biomarkers in current studies are not very specific or sensitive for DbCM. A biomolecule particular to DbCM and less expressed in other pathological scenarios should be the target.

### MicroRNAs

The associations of microRNAs with various diseases have been demonstrated in the past decade. Their role as biomarkers or therapeutics is highly promising. Although much research is progressing in this field, the outcomes have not reached clinical trials as much as those of other conventional drugs. miRNAs have immense potential for clinical diagnostics because of their extensive dysregulation in diseases and high stability compared with mRNAs [153]. These materials are very stable even after many freeze–thaw cycles [154]. These compounds can also be easily obtained from biofluids such as saliva, urine, or blood [155]. Off-target effects of miRNA action are a challenge faced during therapeutic development. Chemical modifications to ensure a longer half-life, reduce immunogenicity, and improve delivery methods should also be prioritized in research [156]. One of the seminal articles that showcases the potential of miRNAs demonstrates miR-21, which is altered in both myocardial tissue and the circulation of diabetic patients. It has a complex role: while it may exert protective effects after cardiac injury, persistent upregulation is associated with maladaptive remodeling, hypertrophy, and fibrosis, positioning miR-21 as both a potential biomarker and therapeutic target in DbCM [157].

### Drug combinations

The synergism of combination therapies has always shown far greater efficacy than that of individual therapies [158]. The combined effect of syringin and tilianin effectively impedes diabetes-induced histopathological changes in DbCM. NLRP3/IL-6/IL-1/TNF-α expression was reduced, and diabetes/hyperglycaemia-induced oxidative stress was suppressed, as evidenced by decreased 8-isoprostane and elevated superoxide dismutase-2 levels in vitro and in vivo [159]. Another combination therapy involving metformin and atorvastatin in palmitate-induced diabetic mice showed superior drug effectiveness compared with individual drug administration. The results revealed significant inhibition of oxidative stress and the expression of inflammatory and apoptotic proteins, e.g., NLRP3, caspase-1, interleukin-1β (IL-1β), Toll-like receptor 4 (TLR4), P-p65/p65, and BAX, in both cardiac tissues and H9C2 cells [160].

### Big data and bioinformatics

Currently, much cardiovascular research is being conducted in animal models or cell lines, which is not a reflection of the actual disease situation in humans. Molecular and genetic profiles from in vitro or animal studies are quite different from those of humans. Conventionally, only heart transplant or postinfarct biopsy samples provide some human-specific results for the disease. The characteristics of the disease in such end phases are drastically different from those in the initial or progressive phase. Therefore, big data from routine hospital examination records or clinical trials can serve as an excellent means to acquire information about the characteristics of the disease and its progression, even in real time. However, ethical guidelines must be followed strictly [161, 162]. The significant challenges are ensuring informed consent, preventing genetic discrimination, and overcoming privacy concerns. Bioinformatic analysis of big data can help understand the patterns and even molecular processes involved in disease development and progression, indicating prospective solutions for wet laboratory studies. High-throughput data submitted in databases are also used to identify potent drug targets via bioinformatic analysis. Endothelin 1 was identified as pivotal in DbCM by integrated bioinformatic analysis [163].

### Regenerative medicine

Cardiomyocytes enter a stage of senescence in DbCM [164]. Therapies that target the clearance of senescent cells are being actively explored [165]. Acute hypertrophy, inflammation, and oxidative stress promote cell apoptosis and necrosis [166]. In this context, cardiac stem cell biology research also has enormous potential. Diabetic rats treated with mesenchymal stem cells presented controlled serum glucose levels and insulin levels and improved cardiovascular performance [167]. The intrinsic regenerative potential of endogenous cardiac stem cells and their ability to be exploited are controversial. However, they have been reported to have replaced ablated cells and revived the damaged myocardium, validating their immense potential [168]. Research aimed at understanding the molecular pathophysiology and intervention at the right time can prevent the progression of DbCM and may help revert cells to their normal physiology.

## Conclusions

This review provides a complete overview of the metabolic conditions of DbCM. The prevalence of the disease, genetics, molecular pathophysiology, current methods of screening, diagnosis, good health practices for prevention, and methods of management are discussed.

DbCM is a cardiovascular disorder caused by pathophysiological changes in the myocardium as a result of diabetes. Screening and diagnosis are not very well established. This review addresses the current methodologies and advancements in structured situation management. The detailed mechanism of pathophysiology is also comprehensively discussed. Hyperglycemia, hyperinsulinemia, glucotoxicity, and lipotoxicity initiate oxidative stress, inflammation, fibrosis, hypertrophy, cardiac remodelling, and eventually HF. Routine examinations, such as blood glucose level, electrocardiogram, and echocardiogram, coupled with MRI, CT, or PET, and currently available biomarkers for cardiac health, help in the better diagnosis of the condition. At present, the disease is managed mainly with antidiabetic medications. More research targeting molecular signalling pathways is needed to develop specific medications. Regular exercise and high roughage content in the diet can prevent or control the onset of the condition. This review provides insights into future research areas for improving the structure of diagnosis and clinical research. Larger cohort studies are lacking for this medical condition, and such clinical data through Big Data can be highly promising for further research developments.

## Data Availability

No datasets were generated or analysed during the current study.

## Abbreviations

DbCM: Diabetic cardiomyopathy
MRI: Magnetic resonance imaging
T2DM: Type 2 diabetes mellitus
ROS: Reactive oxygen species
HF: Heart failure
ECM: Extracellular matrix
RAAS: Renin‒angiotensin‒aldosterone system
BMI: Body mass index
LVDD: Left ventricular diastolic dysfunction
PET: Positron emission tomography
TDI: Tissue doppler imaging
MRS: Magnetic resonance spectroscopy
MMPs: Matrix metalloproteinases
TNFα: Tumor necrosis factor
CGM: Continuous glucose monitoring
GLP-1: Glucagon-like peptide-1
DPP4: Dipeptidyl peptidase 4
SGLT2: Sodium‒glucose cotransporter 2
CANVAS: CANagliflozin cardiovascular assessment
ACCORD: Action to control cardiovascular risk in diabetes
ADVANCE: Action in diabetes and vascular disease: preterax- and diamicron-modified release controlled evaluation
EXAMINE: Examination of cardiovascular outcomes with alogliptin versus standard of care
TECOS: Trial evaluating cardiovascular outcomes with sitagliptin
FIGHT: Functional impact of GLP-1 for heart failure treatment
NIPPV: Non-invasive positive pressure ventilation
LVEF: Left ventricular ejection fraction
ARB: Angiotensin receptor blocker
HRQoL: Health-related quality of life
PROQOLID: Patient-reported outcome and quality of life instruments database
SPECT: Single-photon emission computed tomography
hFABP: Heart-type fatty acid binding protein
TGF-β: Transforming growth factor beta
IFGBP7: Insulin-like growth factor binding protein 7
